# Disability-related-distress in primary school learners with vision impairment due to uncorrected refractive error in KwaZulu-Natal Province, South Africa – a qualitative study

**DOI:** 10.1371/journal.pone.0229108

**Published:** 2020-03-03

**Authors:** Ving Fai Chan, Susanne Singer, Kovin Shunmugan Naidoo

**Affiliations:** 1 Centre of Public Health, School of Medicine, Dentistry and Biomedical Medicine, Queen’s University of Belfast, Belfast, United Kingdom; 2 University KwaZulu Natal, Durban, South Africa; 3 Division of Epidemiology and Health Services Research, Institute of Medical Biostatistics, Epidemiology and Informatics (IMBEI), University Medical Centre of Johannes Gutenberg University, Mainz, Germany; 4 African Vision Research Institute, University of KwaZulu Natal, Durban, South Africa; 5 School of Optometry, University of New South Wales, Sydney, Australia; Università degli Studi di Perugia, ITALY

## Abstract

**Background:**

Uncorrected refractive error (URE) is a major cause of vision impairment among children that impacts negatively on their lives including distresses. We aim to understand the disability-related distress among vision-impaired children due to URE in rural and semi-rural South Africa using qualitative techniques.

**Methods:**

Structured focus groups of children (aged 5–12 years old) with normal vision and vision impairment due to URE from four schools in Pinetown, KwaZulu Natal, South Africa, were performed (four mixed-gender group discussions and eight single gender group discussions). We recruited the study participants after the children underwent standardised vision screening. Criterion sampling was used when selecting study participants. The interviews were transcribed to identify meaning units and broken down to condensed meaning units, which were then grouped into megathemes. Themes were then generated.

**Results:**

Thirteen children with normal vision and 63 children with vision impairment due to URE participated in the twelve focus group discussions with 36 boys (47%) and 40 girls (53%). Twelve themes were generated. The megathemes were Loss of Self Confidence (number of themes (n) = 3), Loss of self-worth (n = 3), Loss of interconnection/ interaction with community (n = 2), Humiliation (n = 2) and Discrimination (n = 2).

**Conclusions:**

We found that vision impairment due to URE can cause distress in different domains in children’s life and further grouped them into different themes. The themes will be used for the development of a tool to assess disability-related distress among children with vision impairment due to URE. We also recommend that distresses caused by URE should be taken into consideration when designing eye care programmes for children.

## Introduction

Distress is an aversive, negative state in which coping, and adaptation processes fail to return an organism to physiological and/or psychological homeostasis”.[1–3] Disability-related distress (DRD) refers to the stress experienced by the individual due to the disability and also influences the relationship between psychological and social processes.[4] Vision impairment (VI) is a form of disability that can have negative effects on an individual and the community.

The prevalence of people living with disability accounts for approximately one-sixth of the world’s population (>1 billion people worldwide).[5] However, this figure may be higher as vision impairment (VI) due to refractive error has been a late entry into the World Health Organization’s numbers. Global estimates also indicate there are 624 million people who are blind or visually impaired because of a lack of spectacles, of which 19 million are children.[5,6] However, currently there is no reliable information regarding the number of children with disabilities. This might be due to differences in definition, or due to the wide range of methodologies and instruments used.

According to the South Africa Census 2011,[7] where the analysis was done on 38,084,876 people older than 5 years old (18,186,962 men and 19,897,914 women), the total number of people living with disability was 2,870,130 (7.5%). Of those, 4.4% were women 3.4% were men. The statistics showed that disability is positively correlated with age; more than half (53.2%) of persons aged 85 and older reported having a disability and compared to the 5–9-year-old age group (10.8%).

Vision is vital for children’s and adolescents’ education and learning, as 80% of learning happens through sight.[8,9] In South Africa[7], the proportion of persons who have difficulty in seeing increases with age (from 3% at age 5–9 years to 49% at age 85 years and older), which is an indication that the aging process has a profound negative impact on the prevalence of VI. However, the impact of VI on children is large. For example, children with severe VI are the most marginalized in accessing primary and secondary education, with children from the mixed-race population being most affected in terms of access to primary education in South Africa.[7]

From the available literature, it was found that children with vision impairment had significantly worse quality of life (QoL) than normal sighted children through focus groups.[10] Uncorrected refractive error (URE) also has been linked to low self-esteem[11] and poor self-concept,[12] and education outcomes[13–15] which are reduced or disappear once refractive correction is made.[16–18]

To the researcher’s knowledge, there is no study on DRD of children related to VI. This descriptive qualitative study explored the VI-related DRD associated with URE in school children in Grades 1 to 5 (approximately aged 6–12 years) in a rural African setting of KwaZulu Natal, South Africa. The results from this study will be used as the evidence from which to develop a tool to measure DRD in primary school learners with VI associated with URE.

## Materials and methods

### Setting and study design

This study was conducted in the four rural public schools of the education district of Pinetown, eThekwini Metro Municipality, in the province of KwaZulu Natal, South Africa. We randomly selected the schools from a list of 23 eligible schools obtained from the Department of Education and ranged in size from 2300 to 6700 pupils (average class size of 50–60 children). We employed criterion sampling to identify the eligible pupils because it provided the best opportunity for the investigator to reach data saturation. The three criteria were Grades (distributed across Grade 1 to Grade 5), visual status (normal versus moderate vision impairment, visual acuity <6/12–6/60 and severe vision impairment, visual acuity <6/60)[17] and gender. We reached the point of no emergence of new data and no new themes (therefore, reached data saturation).

Twelve focus groups were planned. We have chosen a relatively large number of focus groups to reduce the possibility that results would be dramatically affected by a single focus group or methodological choice during coding. We conducted four mixed-gender group discussions and eight single gender group discussions with children aged 5–12 years old. We first contacted head teachers and teachers personally to recruit study participants after the children underwent vision screening. All the children were screened for the first time during our visit. Of those who were identified to have a vision impairment, this was the first time that they had been made aware of their vision impairment. We then conducted short interviews with each potential child’s classroom teacher to ensure the child had no other learning disabilities (such as dyslexia) that could confound the study. A research information pack and consent form together with a formal request letter was sent with eligible children for their parents or guardians. We obtained written informed consent to participate in the research from the guardians or parents prior to seeking assent from each eligible child who had received caregiver’s consent to join the study.

The University of KwaZulu Natal’s Humanities and Social Science Study Ethics Committee (HSS/1061/051D) and the Department of Education (Ref no: 2/4/8/555) approved this study. This paper was prepared according to the Consolidated criteria for reporting qualitative research (COREQ): a 32-item checklist for interviews and focus groups.

The vision screening was done by an optometrist to identify children with and without VI due to URE. Snellen's chart was placed at 6 meters from the child. Vision was screened monocularly and then binocularly. Letters on line 6/12 and 6/24 were isolated. The vision was then screened at 6/12. If a child passed 6/12, a +2.00DS lens was placed in front of the right eye, and the vision was re-screened at 6/24. If the child passed 6/24, s/he was categorized as having refractive error. If the child failed 6/24, s/he had no refractive error. If the child failed 6/12, a pinhole was put in front of the right eye, and the right eye's visual acuity was re-screened at 6/12. If the child passed 6/12, the child was categorised as having refractive error. If the child failed 6/12, ophthalmoscopy was performed on the children. A child was considered passing vision at 6/12 or 6/24 line if the child was able to correctly identify 4 or more letters out of five letters while s/he was considered failing vision at 6/12 or 6/24 line if s/he was able to correctly identify three or less letters out of five letters. The steps were then repeated for the left eye. Finally, the learners’ vision was screened binocularly and recorded. Learners who have vision impairment due to other eye-related co-morbidities were excluded. Learners with learning difficulties were excluded after consulting the class teachers.

### Focus groups discussions (FGDs)

We included school-going children with vision impairment (binocular visual acuity less than 6/12) due to URE and school-going children with no vision impairment. To increase the rigor of our FGDs, we ensured that (1) the groups were relatively small (four to eight participants in each group) to enable the moderator to manage “turn taking” without the use of visual cues; (2) the age and gender distribution of recruited participants reflected the target population (Male: Female ratio of 48% to 52%); and (3) attempts were made to assign participants who know each other to their respective group so that they felt comfortable to participate in the discussion.

Understanding that children as young as 5 years old formed part of the participants, we took steps as recommended by Irwin and Johnson[18] to ensure validity of the focus group discussions. Our interviewers first build rapport with the eligible, consenting participants by having pre-meetings with the children to know them better and to learn about how and where the children preferred the interviews to be conducted. We thus chose a private location for the interview which the children felt comfortable (a classroom far away from other children and teachers).

We then designed our interview tool by adapting the European Organisation for Research and Treatment of Cancer (EORTC) Quality of Life Group Guidelines for Developing Questionnaire Modules. To improve the children’s engagement in the interview process, we included both open-ended and close-ended questions.[19] The focus group questions were developed by two public health optometrists (V.F.C and K.N) and an experienced psychologist (S.S) referencing the European Organisation for Research and Treatment of Cancer (EORTC) Quality of Life Group Guidelines for Developing Questionnaire Modules.[20] We first reviewed the questions from the EORTC Quality of Life Group Guidelines for Developing Questionnaire Modules and determined which of the questions were relevant to our study objectives. We then designed our questionnaire by first dissecting the EORTC Module questions into i) questioning style, ii) the experience and iii) the condition and then framed our questions mirroring this format with an additional “distress experience” component. For example, the EORTC Module question “Can you tell me about the experiences you may have had as a result of your disease” was first broken down to “Can you tell me about” (questioning style), “the experience you may have had” (the experience) and “as a result of your disease” (the condition). We then framed our main question similarly as “Tell me something (questioning style) you would really like to do but are not able to (the distress experience)? Is it due to poor vision (the condition)? How do you feel about it? (the distress experience)? The focus group questions are shown in Table 1.

**Table 1:** Focus group discussion guide.

Key questions	Probes
Can you please tell me what is/are the best thing/s you did in the last two weeks?	Can you tell me more about that?
	Can you think of any additional experiences?’
What do you really like/dislike doing at school?	Can you please tell me, what are the activities you like/dislike the most in your school or classroom? Why?
	How do you feel about it?
How often do you go out to meet your friends, visit relatives, social gatherings and partying?	What do you do with your friends or family in your free time/ for fun or during holidays?
	Can you tell me your hobbies?
	Do you find it difficult to see your friends in school or in playgrounds?
	Do you go shopping? How often? With whom?
	Do you go for social gatherings such as weddings or party?
	Do you go to visit your friends and families in their houses?
	If you don’t do them, is it anything to do with your vision? In what way? Can you give me an example? What makes you think this?
What are the games you play? How do you feel if you are unable to play these games?	Are there games you used to play but stopped? Why?
	Is it anything to do with your vision? In what way? Can you give me an example? What makes you think this?
Tell me something you would really like to do but are not able to? Is it due to poor vision? How do you feel about it?	Why? In what way? Can you give me an example? What makes you think this?

In each focus group, one trained focus group moderators conducted the discussions with a trained person present to take field notes. Two moderators who have more than 2 years conducting interviews with very young children (A.H and M.N) and two trained persons (P.G and Y.L) participated in the study so that the children felt comfortable to share their experiences. To increase the likelihood of freely offered answers, we interviewed the participants without the presence of the teachers or parents. In addition, interviewer training was conducted to minimize the possibility of them leading the child in the conversation that could compromise the integrity of the interview data.

Focus group sessions were 1 to 2 hours long and audiotaped for transcription and analysis. During interviews participants were encouraged to consider all issues that they believed to be relevant to vision impairment due to URE. A constant review of accumulating data ensured the interviews continued until no new issues were raised.

### Data analysis

From a list of responses gathered, we constructed a list of DRD issues that was then given to nine participants (5 children with MVI and 4 children with SVI) for use in an individual Think Aloud debriefing interview to determine what the various issues meant to them, the extent to which they had experienced the problems, further limitations and positive experiences. The debriefing interview was conducted a week after the initial focus group discussions. The DRD issues were developed into short narratives and were read out to the participants and then encouraged to discuss afterwards. This ensured that the items were well understood by the research team and avoid ambiguity in the final list.

Each interview was transcribed verbatim. The interviews were conducted in English. Children were all educated in English in South Africa, and have no challenges interacting fluently in English. All interviewers were bilingual (English and isiZulu). Translators were used only for translating descriptive terms in isiZulu (for example *Mancane*, *Mehlo ekati*, *Shota*) into English. Transcripts were comprehensively reviewed, and meaningful text units were coded to highlight the views and perceptions of the children. Words and phrases (condensed meaning units) from the transcripts were used to link similar statements across focus groups. A database was created consisting of a coded passage of text with its associated condensed meaning unit. We organized and extracted portions of text linked by common condensed meaning units. We then displayed the systematic relationships between coded texts (Sub-category). Using the search function in MS Excel, it was then possible to locate related ideas across the entire focus groups dataset by bringing together strands of data. This process allowed us to explore the data and conceptualize the findings. Children with normal vision were included in the focus group discussions as they served as a control group. Only topics that were mentioned by children with VI but not by children without VI were DRD issue.

Two criteria were used to qualify any topic as an issue. The first criterion was that at least two participants had to have made substantive comments on the topic in a single focus group. This means they did more than just agree with each other and elaborated on the topic based on their experience. The second selection criterion was that the topic was discussed by at least one child in two different groups. Statements about similar issues were condensed under a single heading (theme) and used as examples of features of the issue.

## Results

### Demographic profiles of children interviewed

Vision screening was conducted on children in four primary schools (two rural and two semi-rural). None of the children in the sample had had their vision tested prior to our study. Those who underwent and failed the study screening test were not aware of their vision impairment. Of the 7,693 children screened, 122 (15.9%) children had binocular vision impairment (binocular visual acuity less than 6/12), of which 68 (55.7%) children had URE (eligible participants). Out of the 68 children who failed binocular visual acuity and were categorised as having uncorrected refractive error, 35 had binocular myopia, 27 had binocular hyperopia and 12 had anisometropia. Another 54 (44.3%) failed the test due to other ocular morbidities such as amblyopia, corneal scar or posterior segment morbidities and were excluded from the study).

All but five of the 68 children identified as having VI due to URE invited to participate in a focus group discussion joined one of the groups, resulting in 63 of these children and 13 children without VI being interviewed. Similar proportions of boys (36/76 or 47%) and girls (40/76 or 53%). We further categorized the children with VI as mild VI or MVI (visual acuity <6/12 but ≥ 6/60) and severe VI or SVI (visual acuity <6/60). There were 35 (55.6%) children with MVI and 28 (45.4%) with SVI in the VI sample (Table 2).

**Table 2:** Demography profile of children interviewed in Phase 1.

Group Number	Gender, Number of children		Vision status, Number of children			Grade, Number of children				
	Male	Female	NV	MVI	SVI	I	II	III	IV	V
1	3	4	1	5	1	3	4	0	0	0
2	3	3	0	2	4	0	4	2	0	0
3	4	3	2	2	3	0	1	2	2	2
4	2	5	3	3	1	0	0	1	2	4
5	5	0	0	3	2	5	0	0	0	0
6	6	0	2	4	0	1	5	0	0	0
7	0	6	2	1	3	0	1	4	1	0
8	0	7	0	2	5	0	0	1	6	0
9	8	0	1	3	4	0	0	0	0	8
10	0	4	0	2	2	0	0	3	1	0
11	0	8	0	5	3	0	3	5	0	0
12	5	0	2	3	0	0	0	0	3	2
**Total**	**36**	**40**	**13**	**35**	**28**	**9**	**18**	**18**	**15**	**16**

NV = Normal vision; MVI = Mild vision impairment; SVI = Severe vision impairment

### Disability-related distress identified from focus group discussions

A total of 213 issues were generated from the visually impaired students, of which 96 were included in the theme generation exercise and then clustered into 12 themes and five overarching or megathemes. Of the 117 issues that were omitted, 29 were because children with and without VI mentioned the issue, 37 were because only one person in the same focus group mentioned it, and 51 because it was noted in fewer than two focus group discussions.

When presenting the data, ‘most’ is defined as more than 80% of the respondents, while ‘many” is more than 50% of respondents and ‘some’ is at least 30% of the respondents. Quotes from focus group discussions were denoted with “FGD” while quotes from debriefing de-briefing interview sessions with “DIS”.

#### Megatheme 1: Loss of self-confidence

*Theme 1*: *I feel sad that I cannot participate with friends in games/fun because I cannot see well*. The respondents expressed their sadness by saying that they “feel sad” when they cannot participate with their peers in activities at school and elsewhere because they do not have clear vision. This was particularly articulated in both focus groups with Grade 5 pupils. These activities included sports at school (games) and after school playground activities (fun). The loss of confidence was especially frequently expressed when they believed that “I cannot see well enough”. As one member said:

*“I don’t feel nice because I want to have fun with my friends in the playground*, *but I cannot see well enough”*. (FGD, Female. MVI, Grade 3)

(Group members nodding their heads in unison showing agreement with what was said.)

Students also felt distressed because of the lack of participation. Their loss of confidence was further demonstrated by the respondents in that they perceived that they are as good as their peers, but the fact that their vision is reduced has caused them to reduce their participation time in these activities.

*“I cannot see as well as them (their friends) but I think I am good too*, *especially in soccer*. *I do feel sad sometimes*. *I have one-and-a-half hour of PE and I just play for half an hour*.*”* (Male, SVI, Grade 5)

(Group members giggling)

*Theme 2*: *I feel unhappy that I have to stop playing games because I cannot see clearly*. Unlike Theme 1, where affected children were unable to participate in the activities, this type of distress was described by children who had to end their participation in sports and other play activities because their vision had worsened. A participant expressed:

*“I have to stop playing soccer*. *I cannot see the people and the ball*. *I fell down a lot*. *When I kick the ball*, *I kick air*. *I don’t feel happy because I bought my sport equipment and they are expensive*. *But I can’t play with my friends*.*”* (DIS, Male, SVI, Grade 5)

In another group, the same was felt where he “don’t feel good”, and the loss of confidence was again shown when he “see his friend in the pool, I don’t feel good”. And in some cases, the respondents also experienced injury when taking part in these activities and were regarded as “clumsy”.

*“Yes*. *I don’t play often*. *I used to swim but because I cannot see clearly*, *especially under water*, *I stopped swimming*. *I think I will swim very well if I can see better*. *When I see my friends in the pool*, *I don’t feel good*.*”* (FGD. Female, MVI, Grade 4)

*“I stopped playing jumping castle because I cannot see where I am jumping*, *and I hurt myself when the other children become rough*. *I cried but my mother said I am just clumsy*.*”* (FGD. Male, MVI, Grade 3)

*Theme 3*: *I feel dependent that I need assistance from my teacher/parents/siblings because I cannot see clearly*. The last themes elicited from the domain of loss of confidence was “dependence”. The respondents found that their lack of/reduced ability to do certain tasks because of reduced vision necessitated them seeking assistance from other people, including parents, peers, siblings and teachers. This was particularly experienced by the younger respondents. Two groups from Grade 1 said:

*“My parents will need to help me because I cannot read from the book*. *The words are very small*. *They will help to read it out to me so that I can copy*. *Sometimes my sister helps me too*. *But … (I’m) too slow*.*”* (FGD. Male, MVI, Grade 1)

Another group member followed:

*“I asked (my) sister most of the time*, *but she is also struggling with her own writing (examination)*.*”* (FGD. Female, SVI, Grade 1)

Another member from Grade 1 said the following:

*“(When) I cannot read from the blackboard*, *I will copy from my friend*. *I also ask help from my teacher*. *But there are many friends in the classroom*. *I have to wait until the teacher is free*. *Sometimes I feel shy to ask my teacher*. (FGD. Female, MVI, Grade 1)

#### Megatheme 2: Loss of self-worth

*Theme 1*: *I feel I am not as good as my friends because I cannot see as clearly as them*. The loss of self-worth was evident among students when they compared their skills with those of their peers. Often, they stated they felt they were “not as good as my friends” or “I cannot see as clear as (my friends)”. Many respondents spoke of similar experiences with two respondents saying:

*“I read and write stories and poems*, *but I feel very intimidated because I can’t see as well as them*. *They are more clever because they can see better than me*.*”* (FGD. Female, MVI, Grade 5)

*“(Looking down at the floor) … Sometimes my parents stop me from playing because I cannot see as clear as my friends and they are worried that I will hurt myself*.*”* (FGD. Female, MVI, Grade 3)

*Theme 2*: *I feel lonely that I cannot play with my friends because they can see better than me*. The inability to participate in activities and games with their friends due to their reduced vision had evidently made the respondents feel lonely by saying they were either “uncomfortable, isolated and lonely” or “I’m alone”. The loss of self-worth can be seen when they expressed that “I am not as good (as their friends)”. Frustration was observed in the respondents who had to give up on playing with their friends despite their desire to participate. Two male respondents said:

*“Sometimes if it’s too hot*, *my eyesight becomes worse*. *I cannot see and I cannot play hockey with my friends*. *I feel very uncomfortable*, *isolated and lonely*. *I really want to play with my friends*, *but I am just not as good*. *I cannot see the ball*.*”* (FGD. Male, MVI, Grade 5)

(Other group members laughing)

*“Many times*, *I find it difficult to see my friends*, *especially it’s too sunny*. *I gave up after a while and I’m alone*.*”* (DIS. Male, SVI, Grade 4)

*Theme 3*: *I feel jealous that my friends do better than me because I cannot see clearly*. The respondents also expressed feelings of jealous, grumpy or envious of their peers who out-performed them because of better vision. For example, respondents from Grade 5 and Grade 4 said that:

*“I want to compete with my friends*. *I feel envious that they always do better because they can read faster than me*. *They can run faster*. *They see better*. *But my teachers give me assistance*. *But I feel like I am also at the same level with my classmates*.*”* (FGD. Male, SVI, Grade 4)

The respondents felt this based on comparing themselves with their peers regarding the ability to carry out the same activities, or it was caused by the fact that they were “not chosen” to perform certain activities. Respondents also mentioned that:

*“I am grumpy and envious because they see well*, *they are always chosen by the teachers to do class activities*.*”* (FGD. Female, SVI, Grade 5)

*“I cannot pay full attention in whatever I am doing*. *I feel jealous of ______ because he is always doing so well*. *I think it’s my small eyes*. (FGD. Male, MVI, Grade 1)

*(Another member pulling his eyes up to demonstrate the inability to see clearly while other members started giggling*)

#### Megatheme 3: Loss of interconnection/interaction with community

*Theme 1*: *I do not go for outside school activities with my friends as much as I wish because I cannot see very well*. There were many respondents who were unable to participate in activities outside of school, such as “going out with their friends”. The loss of interaction was shown when the respondents felt that they were a burden because they had to be “taken care of” and their friends “have to let me win”. For example:

*“I don’t go out with them (my friends)*. *They feel they have to wait for/ take care of me/ I’m slow*.*”* (FGD. Female, SVI, Grade 1)

*“I’m not invited because they have to let me win*.*”* (FGD. Male, SVI, Grade 1)

*Theme 2*: *I do not go to family/friends’ parties because I cannot see very well*. The respondents also noted that they were unable to participate in parties organized by friends, family or relatives for fear by others that the students might get hurt or lost if in unfamiliar places. Their participation in parties is also limited as the respondents were dependent on a chauffeur, such as an older brother. A respondent from Grade 5 said that:

*“I like visiting friends and relatives*. *We walk to their places sometimes*. *But because I cannot see clearly*, *my mother does not allow me to go alone*, *unless my brother goes with me*. *But he is older*. *He is in Grade 12*. *He needs to study*. *So I stay at home until he is free*.*”*(FGD. Male, MVI, Grade 5)

And another respondent also said that:

*“I rarely go out to family gatherings*. *I am slow*. *I always get lost*. *I am not sure but I think it’s because I cannot see clearly and I cannot recognise the road*.*”* (FGD. Male, SVI, Grade 4)

#### Megatheme 4: Humiliation

*Theme 1*: *I am excluded from games because I cannot see clearly*. While the respondents did not report obvious suspicion and physical abuse (fight) from their friends and the community, they did experience humiliation in the form of “exclusion”. Sometimes this was because they were seen as a burden to bigger group as they “made them lose in the competition”. Some respondents also have lost interest in the activities they were participating in or intended to participate in. Respondents said that:

*“They exclude me from the team because I cannot see clearly*. *I make them lose in the competition*.*”* (FGD. Male, MVI, Grade 5)

*“Sometimes they ask me to sit aside in the playground*. *I don’t want to do it anymore*.*”* (FGD. Female, SVI, Grade 2)

*Theme 2*: *I am called names because I cannot see very well*. One of the humiliations felt by the respondents was being given nicknames such as *“Four eyes”* (Male, MVI, Grade 4), *“Mancane”*, which means small (Female, MVI, Grade 4), *“Mehlo ekati”*, which means cat eyes (Female, MVI, Grade 3) and *“Shota”*, which means short (Male, SVI, Grade 2). This was reported by most of (8 out of 10) the focus groups.

#### Megatheme 5: Discrimination

*Theme 1*: *I felt left out that I am asked not to participate because I cannot see clearly*. The respondents particularly felt “left out” and “ignored” when asked to “sit on one side” as a result of their reduced ability to perform certain tasks due to their reduced vision. A respondent mentioned that they have “the right to play, too”, indicating some degree of discrimination being felt in those circumstances.

*“Sometimes*, *I felt left out when playing because I cannot see clearly*. *But I have the right to play too*! *They just ignored me*.*”* (FGD. Male, MVI, Grade 3)

*“I don’t like it when my class teacher asks me to sit at one side because I cannot see the blackboard clearly*. *They beat me sometimes*. *Because I am slow in reading and copying*. *I cannot see clearly and (cannot) read fast*.*”* (FGD. Female, MVI, Grade 2)

*Theme 2*: *I feel sad that I am asked not to participate because I cannot see clearly*. Similarly, the respondents spoke about the inability to read or play certain sports, such as “cannot read the notes” or “I cannot catch the ball” due to their reduced vision made them “sad and cried” or “felt very bad”. This feeling was felt from being told “not able to join” and “not chosen”. For example:

*“I want to sing in the choir*. *I was told that I am not able to join because I cannot read the notes from the blackboard*. *I was very sad and I cried*. *I tried again the next year*.*”* (FGD. Female, MVI, Grade 4)

*“(I) went for sports day but never get chosen for soccer team*. *My brother plays school team*. *But I am always not chosen*. *They say I cannot catch the ball*. *I know I’m short but I also cannot see the ball*. *I felt really bad and I told my mum*. *She said try other sports but I like soccer*.*”* (FGD. Male, SVI, Grade 3)

## Discussion

Vision plays a critical role in a child’s life in both their developmental and educational learning.[8] The inability to see clearly can cause a delay in achieving developmental milestones, such as effective communication and social skills acquisition. The delay in developing these life skills, which are extremely essential for participation in group activities (such as games), can consequently cause the children with VI to lag behind their peers in both curricular and extra-curricular activities.[21] Miller,[22] while trying to analyse and adapt teaching techniques according to the social skills developmental stage of children with VI, found that the lack of social skills and social activities among children with VI is exceptionally detrimental because these are closely linked to self-concept and self-esteem and can therefore negatively impact the overall well-being of the child. He further emphasizes that social skills lead to social learning, which has its foundation in good learning and task development skills that many children with VI have not developed.

One important and preferred method of developmental learning is through games or fun activities because they involve exploration, organization, and synthesis of information while interacting with other children. When children are unable to do so due to their inability to see clearly, they feel discouraged and this physical limitation impacts negatively on their curiosity to learn and explore.[23] Seligman,[24] in an animal study, described this as “learned helplessness” and the exhibition of passive and helpless behavior can be expressed simply as “unhappiness” by children. Our study showed that the children with VI stopped playing games as they found effective participation extremely difficult. A short child may not have been teased for being short during play if he could see and play as well as the peers.

All the children with VI in the classroom had not been identified as having this difficulty prior to our study, and hence at least some of their educational needs were likely to not have been met. For example, the ability to read and copy from the chalkboard was greatly reduced and the children placed great reliance on their friends sitting next to them. Our finding agrees with Margalit’s study,[25] which compared the leisure activities of 51 children with cerebral palsy when compared to those of physically healthy controls. The study found that children with cerebral palsy were also more dependent on others. While the nature of disability between the current study and Margalit’s study is different, there seemed to be a similarity in their feeling of dependence. This feeling of dependence, as described by Kitchin,[26] can be a distress that is internalized as well as lead to marginalization.[26–28]

While some children expressed their need for more assistance from their teachers, Watson et al.[29] warned that assistance should be given cautiously because it may emphasize that children with disabilities need “extra help” and thus portray the children as “different” from or weaker than their peers. This can further create negative identities among the children.

Furthermore, environmental factors such as lack of facilities, limitation of play space and lack of physical assistance[30–32] may also discourage children with VI from participating in games that they used to play. In mainstream public schools, infrastructure is designed according to the needs of “average” children. Children with disabilities may face restrictions in these physical environments and find themselves unable to integrate into the schooling community.

The feeling of being “not as good as my friend” was described as an internalized representation. This was also observed by Holt[33] as existing in children with learning disabilities. The study by Holt was undertaken to understand what “disabled” children experience and how they performed while going to mainstream schools in England. The study found that the children perceive themselves as “not good” at many subjects taught in schools. Holt argued that this may be due to the high expectations of adults towards the children. This demonstrated that to improve classroom participation of children with VI, interventions should be implemented beyond the four walls of the education institution and include the parents, family, the community and the society.

Another important observation in our study was that children were age-organized into different grades. While it is common practice in school environments with high student-teacher ratios to have some form of group organization to meet societal expectation, children with VI, or any disability, may not have the same level of competency as children with NV even if they are of the same age. This puts children with VI at a distinct disadvantage socially and academically when compared with their classmates.

The children enrolled in our study reported feeling lonely due to their inability to participate in activities with friends. Feeling lonely has been described as a “children factor” that is caused by lack of confidence in social activities such as playing with friends or feeling awkward or self-consciousness among the children with VI. This, in turn, can cause friendlessness and limited empathic abilities (the ability to share feelings together) because the child's emotional, behavioural and social spheres become affected.[34–37] Loneliness can be a threat to a child’s development as supportive relationships are critical for developing social relationships, and help the child to participate in daily activities.[37–39]

The feeling of jealousy was expressed by a few children with VI who indicated they frequently were punished by teachers because they were “slower” than their peers. This was seen as a “hidden rule”[40] (rewarding pupils who perform according to expectations and penalize those who perform below the set standards). When observing their peers being praised by teachers for academic achievement, and thus reaffirming their peers’ identity as being a success,[41] children with VI felt they were inferior students and people.

The children with VI described feeling excluded and stigmatized at school, especially with regards to group activities. Their interaction with children without VI became challenging when there was an obvious difference in their ability to perform in group activities requiring good vision. Children with VI were seen as a burden to their team and blamed as the reason for a team’s under-performance. They were often excluded from team activities, a phenomenon observed in other studies.[41,42] Dear et al.,[42] in their meta-analysis of 44 hierarchy studies, also observed that children with learning disabilities are usually more often excluded from children’s cultures than their healthier peers. The failure to participate in leisure and recreational activities such as games then becomes a predictor of reduced life satisfaction and well-being later in life[43,44] because they are restricted in their ability to develop social, intellectual, emotional and communication skills.

In our study, pupils with VI were often given derogatory nicknames in their schools. This was also recognised by Law and Dunn[45] who described that disabilities restrict the children to their constrained environment, causing them to be unable to participate in community activities, thus preventing them from integrating socially. Derogatory nicknames were often given to the children with disabilities and served as a reminder of why they are not integrated into the broader society, often due to negative community attitudes towards children with disability.[33,46,47]

As mentioned previously, children with VI are often asked not to participate in group activities. Brown and Gordon[48] perceived this as a society’s distorted reaction towards disability. In their study documenting daily activities of children with versus without disabilities in a New York hospital setting, they found that children with disabilities spent more time engaging in dependent and quiet activities compared to socially engaging activities. The feeling of being “left out” may further make the children feel more handicapped because they are prevented from accumulating experience from these group activities. Their inability to participate in group activities prohibits them from acquiring a broad range of life skills, feeling incompetent and self-determination becomes weak. This was also described by Fraser[49] who further argued that to overcome the impact of disabilities, intervention should target the contributing factors and not the impairment only. The inability of the children with disability to participate in meaningful activities decreases their quality of life.[50–52]

### Limitations

Themes identified in this study are based solely on the views of school-going children and not teachers or parents who might observe the impact of VI on school-aged children’s activities and social integration. Thus, we may have overlooked some issues that could have been observed by teachers and parents and may not have been reported by the children. However, these DRD issues are to be used as the basis to develop a DRD measurement tool that is to be administered to school-going children. In our study, a topic to be considered important by two data analysts to be regarded as significant because this criterion was used to define a DRD issue. This analysis process may have lost some richness in our findings because thoughts raised only once are of utility in understanding the child’s DRD experience. We also assumed that school-going children may not relate to issues identified by parents and teachers, thus the inclusion of these issues would reduce the ceiling and floor effect of the tool.

Our screening protocol to identify children with refractive error may have missed a small number of children with low to medium hyperopia We did not conduct cycloplegic refraction in this study because most parents would not allow their children to undergo cycloplegic refraction (determined in our feasibility study)- leading to low participation rate. Parents were concerned that cycloplegia could cause many side effects and were fearful that these side effects could be permanent. We could not perform any clinical assessment on site because the Health Professional Council in South Africa stipulated that no onsite assessment should be carried out during screening because the school environment does not meet clinical standards. Consent provided by parents was only for the children’s vision to be screened with gross examination with pen torch. We thus adapted the school screening guidelines recommended by IAPB by adding a +2.00D test to categorise the children into hyperopia.

In the current school system, other learning disabilities could have been missed and affected the results because very seldom proper evaluation of children are done especially in rural areas. However, even if this was the case, the impact would have been only on a small subset.

As our study objective is to understand the disability-related distress among children with vision impairment due to uncorrected refractive error, we only included children who failed binocular visual acuity testing. We hypothesized that those who passed binocular visual acuity testing (even though they had a monocular vision impairment), could function normally and therefore, faced minimal distress.

## Conclusion

Children have to be treated as whole human beings. Focusing only on eye and vision function, without taking into consideration the psychological, social and emotional well-being of the children, makes it challenging to integrate child eye health with child health. IN this study, we found that vision impairment due to URE can cause distress in different domains in children’s life and further grouped them into different themes. The themes will be used for the development of a tool to assess disability-related distress among children with vision impairment due to URE. We also recommend that distresses caused by URE should be taken into consideration when designing eye care programmes for children.

## Supporting information

S1 Database (XLSX)
